# Noisy Preferences in Risky Choice: A Cautionary Note

**DOI:** 10.1037/rev0000073

**Published:** 2017-06-01

**Authors:** Sudeep Bhatia, Graham Loomes

**Affiliations:** 1 Department of Psychology, University of Pennsylvania; 2 Behavioural Science Group, Warwick Business School, University of Warwick

**Keywords:** decision making, noise, risky choice, prospect theory

## Abstract

We examine the effects of multiple sources of noise in risky decision making. Noise in the parameters that characterize an individual’s preferences can combine with noise in the response process to distort observed choice proportions. Thus, underlying preferences that conform to expected value maximization can appear to show systematic risk aversion or risk seeking. Similarly, core preferences that are consistent with expected utility theory, when perturbed by such noise, can appear to display nonlinear probability weighting. For this reason, modal choices cannot be used simplistically to infer underlying preferences. Quantitative model fits that do not allow for both sorts of noise can lead to wrong conclusions.

Research on risky choice has relied heavily on the use of deterministic utility maximization models, such as expected utility theory (EUT; Von Neumann & Morgenstern, 2007) and cumulative prospect theory (CPT; Kahneman & Tversky, 1979; Tversky & Kahneman, 1992). When their functional forms are specified and parameterized, these models make precise quantitative predictions. However, they fail to capture an important aspect of actual choice behavior: namely that choice is stochastic, so that decision makers may respond differently when given exactly the same choice problem on more than one occasion within a short space of time (see Mosteller and Nogee, 1951, for early evidence; and Luce and Suppes, 1965, for a discussion; Rieskamp, Busemeyer, and Mellers, 2006; Wilcox, 2008; and Marley and Regenwetter, 2016, provide more recent reviews of key issues).

There are two main ways in which stochasticity in choice has been accommodated. One approach involves allowing for some error in selecting the utility-maximizing option, with the overall choice probabilities being determined by the relative utilities of the gambles in consideration (e.g., Luce, 1959; McFadden, 1973; Thurstone, 1927). An alternative approach allows the parameters of an individual’s utility function to vary from one moment to another, with choice probabilities being determined by the likelihood of one gamble having a higher utility than the other at a particular moment (e.g., Becker, DeGroot, & Marschak, 1963; Loomes & Sugden, 1995). The first approach can be interpreted as involving response noise: that is, noise that applies after the utilities for the gambles have been determined according to some model and compared. In contrast, the second approach involves noise in the utility generation process itself: that is, variability in the decision maker’s underlying preferences. This note warns that unless the analysis of risky choice data considers both forms of noise, false conclusions may be drawn about people’s underlying preferences.

## Risky Decision Models

We can write two-outcome risky gambles as *X* = (*x*_1_, *p*_1_; *x*_2_, *p*_2_), so that *X* offers payoffs *x*_1_ and *x*_2_ with probabilities *p*_1_ and *p*_2_, with *p*_1_ + *p*_2_ = 1. Using a power function formulation for the value of any payoff *x* and Prelec’s (1998) one-parameter probability weighting function, the EUT or CPT utility of *X* can be written as^1^:

$$U(X|\alpha ,\gamma )=\pi (p_{1})\;\cdot \;{x_{1}}^{\alpha }+(\text{1}-\pi \left(p_{1}\right))\;\cdot \;{x_{2}}^{\alpha }$$ 1

where *x*_1_ ≥ *x*_2_ ≥ 0 and π(*p*_1_) = $e^{-(-\ln p_{1}{)}^{\gamma }}$ and where we restrict α > 0 and γ > 0. CPT allows both α and γ to vary. When γ = 1, we have EUT as a special case of CPT. We obtain expected value when both α = 1 and γ = 1. When α < 1, concave value functions produce risk averse choices under EUT, whereas α > 1 corresponds with risk seeking. When γ < 1, we have overweighting (underweighting) of small (large) probabilities, with γ > 1 producing the opposite.

Aside from error, EUT and CPT models assume that *X* is always chosen over *Y* if *U*(*X*|α, γ) > *U*(*Y*|α, γ). To accommodate probabilistic choice data, some variability must be incorporated. One approach has been to assume that there is some error in the response such that the probability of selecting gamble *X* over *Y* is given by some increasing function of *U*(*X*|α, γ) – *U*(*Y*|α, γ). In many studies, this is implemented either by the logit model (Luce, 1959; McFadden, 1973) or by the probit model (Thurstone, 1927). In the binary choice case, both models can also be interpreted as involving an additive error ε, with E[ε] = 0, so that the probability of choosing *X* is the probability that *U*(*X*|α, γ) – *U*(*Y*|α, γ) + ε > 0 (Yellott, 1977).^2^ We refer to variability added to the core utilities as ‘response noise.’

A second way of modeling stochastic choice involves allowing the parameters of decision makers to fluctuate (Becker et al., 1963; Loomes & Sugden, 1995). For example, suppose α = α* + η_α_ and γ = γ* + η_γ_, where η_α_ and η_γ_ are symmetric random variables with E[η_α_] = E[η_γ_] = 0. η_α_ and η_γ_, and thus α and γ, *v*ary from trial to trial (but not between options in a given trial). Because the expected values of α and γ are E[α] = α* and E[γ] = γ*, α* and γ* characterize the central tendency of a decision maker’s underlying preferences.^3^ We refer to such variability in model parameters as ‘preference noise.’

Preference noise and response noise can coexist. For example, if we assume that response noise is given by the logit model, the choice probability of *X* generated by a particular realization of η_α_ and η_γ_, and subsequently α = α* + η_α_ and γ = γ* + η_γ_ is:

$$\text{Pr}\left[X\,\;\,\,\text{chosen}\right]=\frac{e^{\theta \cdot U\left(X|\alpha ,\gamma \right)}}{e^{\theta \cdot U\left(X|\alpha ,\gamma \right)}+e^{\theta \cdot U\left(Y|\alpha ,\gamma \right)}}$$ 2

Note that η_α_ and η_γ_ are random variables so that the overall choice probability of *X* over *Y* in any given trial can be obtained by calculating the expectation of Pr[*X* chosen], given the distribution of η_α_ and η_γ_. θ is a parameter that is inversely proportional to the degree of response noise in the choice process.

Theoretically there are many reasons to assume both preference noise and response noise. Preferences (and in turn, the parameters that characterize these preferences) can fluctuate, reflecting variations in attitudes, noise in the process of deliberation, or changes in affective states. Additionally, there may be numerous factors (e.g., computational mistakes, inattention to some elements of the decision) that potentially overturn the decision maker’s underlying preference, with the frequency of such response errors depending on the relative desirability of the utility maximizing option.

Empirically the assumption of both preference noise and response noise can offer a more adequate account of choice data than each of these assumptions alone. For example, allowing for only response noise in the above framework generates much higher frequencies of violations of transparent dominance than are generally observed, whereas allowing for only preference noise leads to the prediction that dominance is never violated at all, contrary to the evidence (Loomes & Sugden, 1998; also see Butler, Isoni, & Loomes, 2012 and Busemeyer & Townsend, 1993 for a related effect). Likewise, EUT and CPT have been fit to choices elicited at different points in time (Glöckner & Pachur, 2012; Zeisberger, Vrecko, & Langer, 2012). The fits assume only response noise, but the best-fit parameters also exhibit variability, with decision makers’ estimated preferences at one point in time being correlated with, but not identical to, their estimated preferences at a different point in time. Related work has fit EUT and CPT models, permitting both response and preference noise, and has found that both types of noise are necessary for the best quantitative fits (Blavatskyy & Pogrebna, 2010; Loomes, Moffatt, & Sugden, 2002).

## Qualitative Inferences

### Noisy Risk Attitudes

It might be thought that if both types of noise are unsystematic (symmetrically distributed around zero), modal choices can be used to make inferences about underlying preferences. This section tests that intuition and shows that it is incorrect.

Consider the choice between a risky gamble *X* offering a 50% chance of obtaining $10 and a 50% chance of obtaining $0, and its safe expected value equivalent *Y* offering $5 with certainty. Assume that a decision maker’s central tendency is described by the power form of EUT (i.e., γ = 1 in Equation 1) and that choices display both preference and response noise as specified in Equation 2. Suppose also that response noise involves θ = 1 and that preference noise involves η_α_ distributed uniformly in the interval [−0.5, 0.5]. Let α* = 0.9 so that modal underlying preferences are risk averse. However, when response noise is added, Pr[*X* chosen] = 0.53 > 0.5. Thus, despite underlying preferences predominantly favoring *Y*, the decision maker chooses the riskier *X* more frequently than *Y*.

This mismatch between underlying preferences and observed choices happens because of the nonlinearity of utility differences in α. The probability of choosing *X* is an increasing function of *U*(*X*|α, 1) – *U*(*Y*|α, 1) = 0.5 · 10^α^ – 5^α^. For the range of α, we are considering, E[*U*(*X*|α, 1) – *U*(*Y*|α, 1)] > 0, resulting in a higher choice probability of *X*, despite the fact that α* < 1 and that *U*(*X*|α*, 1) – *U*(*Y*|α*, 1) < 0.

**Figure 1:**
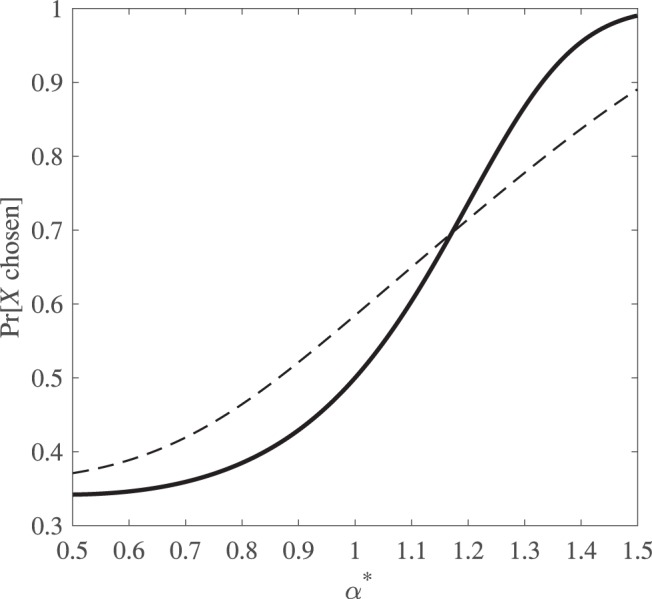
The probability of choosing a risky gamble *X* over its expected value *Y* for varying values of α*, plotted with only response noise (solid line) and with both response and preference noise (dashed line). Here we can observe a higher choice probability of *X* over *Y* for some values of α* < 1 in the presence of preference noise.

The point is expanded upon in Figure 1. We plot the probability of choosing *X* = ($10, 0.5; $0, 0.5) over *Y* = ($5, 1) according to power function EUT with only response noise (implemented via a logit function with θ = 1), and we compare that with the case in which preference noise (with η_α_ distributed uniformly in the interval [−0.5, 0.5]) is combined with the same specification of response noise. The first model entails Pr[*X* chosen] less than, equal to, or greater than 0.5 according to whether α* is less than, equal to, or greater than 1, as shown by the solid line in Figure 1. However, in the case when α is variable—shown by the broken line in Figure 1—there is a range of values of α* between 0.87 and 1 where Pr[*X* chosen] > 0.5. Over this range, the decision maker’s expected modal choice suggests risk seeking, whereas the central tendency of underlying preferences, represented by α*, suggests risk aversion or risk neutrality. In short, when both preference noise and response noise are present simultaneously, we cannot use modal choices to make reliable inferences about the decision maker’s risk attitude.^4^

### Noisy Probability Weighting: The 4-Fold Pattern

We now turn to cases in which probabilities may be transformed nonlinearly. We use the single parameter Prelec (1998) formulation outlined in Equation 1, but other transformation functions (Gonzalez & Wu, 1999; Tversky & Kahneman, 1992) could be used without altering the essential conclusions. When γ < 1, this function overweights low probabilities and underweights high probabilities. Such an inverse-S function is crucial to Tversky and Kahneman’s (1992) account of the 4-fold pattern of risky choice.

In the positive domain considered here,^5^ the 4-fold pattern entails a risky gamble being chosen over its expected value when the probability of the higher payoff in the risky gamble is small but the opposite pattern when the probability of the higher payoff in the risky gamble is large. Thus, in the choice between a risky gamble *X*^I^ offering a 1% chance of obtaining $10 and a 99% chance of obtaining $0 and its safe expected value equivalent *Y*^I^ offering $0.10 with certainty, decision makers typically choose *X*^I^. In contrast, in the choice between a risky gamble *X*^II^ offering a 99% chance of obtaining $10 and a 1% chance of obtaining $0, and its safe expected value equivalent *Y*^II^ offering $9.90 with certainty, decision makers typically choose *Y*^II^.

**Figure 2 fig2:**
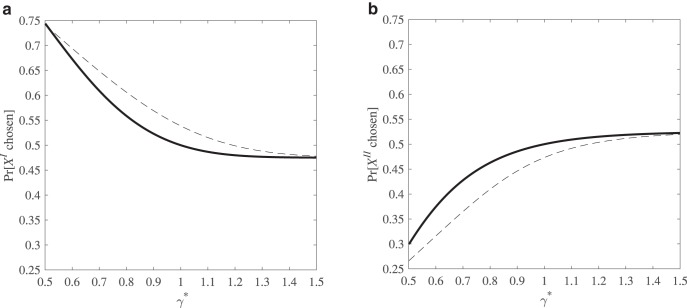
a and b. The probability of choosing a low-probability risky gamble *X*^I^ over its expected value *Y*^I^ (left panel) and the probability of choosing a high-probability risky gamble *X*^II^ over its expected value *Y*^II^ (right panel) for varying values of γ*. These figures are plotted with only response noise (solid line) and with both response and preference noise (dashed line).

Consider a setting with both response and preference noise. Let α = 1 so that the value function is linear, and allow noise only in the γ parameter, with η_γ_ being distributed uniformly in the interval [−0.5, 0.5]. For response noise, use the logit function with θ = 1 as in the previous section. Figure 2a shows the probability of choosing *X*^I^ over *Y*^I^ and Figure 2b shows the probability of choosing *X*^II^ over *Y*^II^. As shown by the solid line, a model with response noise only and with γ* = 1 entails for both pairs a 0.5 chance of choosing each option. For all γ* < 1, the risky option is the modal choice in Figure 2a, whereas the sure amount is the modal choice in Figure 2b. However, when γ exhibits preference noise, the effect—as shown by the broken line—is to shift the path up in Figure 2a and down in Figure 2b: the combination of preference and response noise increases the choice probability of *X*^I^ over *Y*^I^ and of *Y*^II^ over *X*^II^ for all γ* considered.[Fig-anchor fig2]

At the point at which α = 1 and γ* = 1—that is, in the case in which the underlying preference entails a risk-neutral expected utility maximizer—the modal choices exhibit the mixed attitude to risk typical of CPT with γ < 1. Indeed, there is a range of γ* between 1 and 1.15 for which the decision maker’s expected modal choices generate a preference for *X*^I^ over *Y*^I^ and for *Y*^II^ over *X*^II^, a behavioral pattern associated with the overweighting of small probabilities, whereas that range of γ* represents an underweighting of small probabilities. Again, we cannot use modal choices to infer probability weighting if preference and response noise are present simultaneously.^6^

### Noisy Probability Weighting: The Common Ratio Effect

The probability weighting transformation assumed by CPT also enables it to account for the common ratio effect (Kahneman & Tversky, 1979). The classic common-ratio case involves choices between two pairs of lotteries. One pair offers a gamble *X*^III^ = (*x, p*; 0, 1 − *p*) versus *Y*^III^ = (*y*, 1), where *p* is typically around 0.8 and where *y* is near the expected value of *X*^III^. In the example we consider, our scaled-up pair is a choice between a gamble *X*^III^ offering an 80% chance of obtaining $10 and a 20% chance of obtaining $0 and its expected value equivalent *Y*^III^ offering $8 with certainty. For such a pair, decision makers typically choose the sure option *Y*^III^.

The second pair involves scaling down the probabilities of the positive payoffs in the first pair by some factor λ and correspondingly increasing the probabilities of 0 in both options to give a choice between *X*^IV^ = (*x, λp*; 0, 1 − λ*p*) and *Y*^IV^ = (*y*, λ; 0, 1 − λ). Letting λ = 0.25 gives *X*^IV^ offering a 20% chance of obtaining $10 and an 80% chance of $0 versus *Y*^IV^ offering a 25% chance of obtaining $8 and a 75% chance of $0. In such scaled-down pairs, decision makers choose the riskier option *X*^IV^ much more frequently. This is inconsistent with EUT, which assumes that preferences are linear in probabilities. In a deterministic world of EUT maximizers, whatever proportion of the sample chooses *X*^III^ in the first pair should also choose *X*^IV^ in the second pair.

**Figure 3 fig3:**
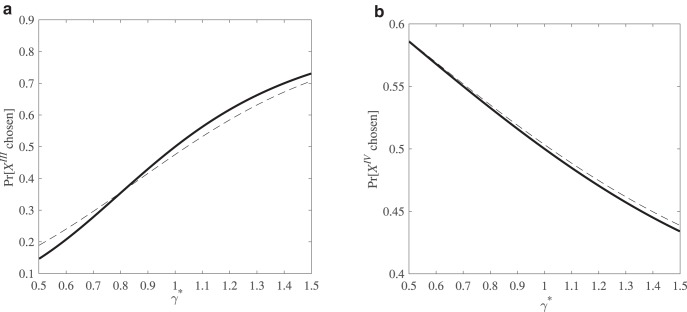
a and b. The probability of choosing a scaled-up risky gamble *X*^III^ over its expected value *Y*^III^ (left panel) and the probability of choosing a scaled-down risky gamble *X*^IV^ over its expected value equivalent safe gamble *Y*^VI^ (right panel) for varying values of γ*. These probabilities are plotted with only response noise (solid line) and with both response and preference noise (dashed line).

The change in modal choices often found in the data can be accommodated by CPT with γ < 1. This is illustrated by the solid lines in Figures 3a and 3b in which we fix α = 1, assume a logit noise term (with θ = 1) and let γ* range between 0.5 and 1.5. Over the range γ* < 1, a model with only response noise entails that *Y*^III^ is the modal choice in Figure 3a, whereas *X*^IV^ is the modal choice in Figure 3b.[Fig-anchor fig3]

Now allow response and preference noise to coexist and let η_γ_ be distributed uniformly in the interval [−0.5, 0.5]. This produces a shift in the choice probabilities, with an increase in the choice probability of *Y*^III^ over *X*^III^ and of *X*^IV^ over *Y*^IV^ for γ* in the neighborhood of 1. Thus, even when α = 1 and γ* = 1, the modal choices exhibit the reversal observed in many experiments, with a preference for *Y*^III^ in the scaled-up pair but a preference for *X*^IV^ in the scaled-down pair. Here, too, modal choices cannot be used to infer the underlying preference of decision makers: the common ratio effect can be generated by risk neutral expected utility maximizers.^7^

## Quantitative Inferences

### Recovering Risk Preferences

In this section, we explore the effects on quantitative model fits if preference noise is neglected. Particularly, we simulate the choices of decision makers when the two forms of noise are present simultaneously and then examine what happens if we attempt to recover best-fit parameters with only response noise modeled.

We begin with EUT, considering only variability in the parameter α (i.e., restricting γ = 1). We perform two sets of simulations: one in which α is deterministic, with α = α*, and another in which α is probabilistic, with α = α* + η_α_ and η_α_ distributed uniformly in the interval [−0.5, 0.5]. We vary α* in the range [0.5, 1.5], and for each value of α*, we simulate the corresponding EUT model on Stott’s (2006) gamble pairs.^8^ The best-fit values of α for the choices generated by the deterministic α simulation and probabilistic α simulation are then recovered. Our first recovery involves α generated by the probabilistic α simulation, under the (incorrect) assumption that α is deterministic. This recovery can help us establish the degree to which parameter recovery is biased when the data-generating model involves both sources of noise but the fitted model involves only response noise. The second recovery involves α for the choices generated by the probabilistic α simulation under the (correct) assumption that α is probabilistic.^9^ The third recovery involves α for the choices generated by the deterministic α simulation under the (correct) assumption that α is deterministic. These latter recoveries help us evaluate the efficacy of parameter estimates when the underlying model is correctly specified.^10^

**Figure 4 fig4:**
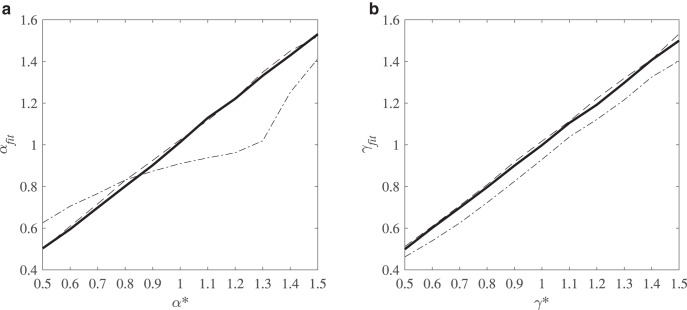
a and b. Median recovered values of α, α_*fit*_, plotted against α* (left panel) and median recovered values of γ, γ_*fit*_, plotted against γ* (right panel). The first parameter recovery (dotted-dashed line) involves a data-generating model with both response and preference noise and a misspecified fitted model which assumes only response noise. The second parameter recovery (dashed line) involves a data-generating model and correctly specified fitted model with both response and preference noise. The third parameter recovery (solid line) involves a data-generating model and correctly specified fitted model with only response noise.

Figure 4a displays the median recovered α, from now on referred to as α_*fit*_, for each value of α* for each of the three parameter recoveries. It shows that α_*fit*_ is very close to the corresponding α* when the fitted model is correctly specified. In contrast, α_*fit*_ differs quite significantly from α* for the first recovery in which the fitted model incorrectly assumes deterministic α when α is in fact probabilistic. In that case, the recovered parameter values are systematically biased. Particularly, α_*fit*_ > α* when α* is small but α_*fit*_ < α* when α* is large. Thus, highly risk averse decision makers appear less risk averse than they actually are, whereas risk-neutral (α* = 1) and some risk-seeking (α* = 1.1, 1.2) decision makers actually appear to be risk averse.[Fig-anchor fig4]

### Recovering Probability Weighting Parameters

To examine probability weighting biases, we perform two sets of simulations: one in which γ is deterministic, with γ = γ*, with only response noise; and another in which γ is also probabilistic, with γ = γ* + η_γ_ and η_γ_ distributed uniformly in the interval [−0.5, 0.5]. For both sets of simulations, we take γ* values in the range [0.5, 1.5]. To focus on probability weighting biases, we fix α at 1. All other aspects of our parameter recovery exercise are identical to those in the previous section.

Figure 4b displays the median recovered γ, from now on referred to as γ_*fit*_, for each value of γ* for all three sets of recovered parameters. Again, the fitted value is very close to γ* in the second and third recoveries, when the fitted models are correctly specified. In contrast, γ_*fit*_ differs quite significantly from γ* for the first (misspecified) recovery. Moreover, the recovered parameter values are systematically biased, with γ_*fit*_ < γ* for all the values of γ that we consider, for the first recovery. Decision makers without any central tendency disposition to transform probabilities (γ* = 1) appear to overweight small probabilities (with γ_*fit*_ < 1) if their choices are fit with the assumption that there is no parameter variability.

The supplemental materials (http://dx.doi.org/10.1037/rev0000073.supp) show that these parameter recovery biases also emerge when both α and γ are recovered together.

## Correlates of Risk Preference

If it can be unsafe to infer an individual’s underlying preferences from modal choice patterns or from quantitative fits of models that only allow for response noise, it may also be unsafe to infer differences in preferences between different groups of individuals or between individuals in different experimental conditions.

Between them, the disciplines of psychology, neuroscience, and economics have produced a large number of studies examining the relationship between risk preference and a wide variety of social, biological, cultural, cognitive, emotional, and neural variables. Much of this work makes the implicit or explicit assumption that differences in modal choice probabilities between different experimental or demographic groups reflect differences in underlying value functions and/or probability weighting preferences.

For example, based on choice proportions, men are considered to be more risk seeking than women (Charness & Gneezy, 2012), a tendency that is amplified by contextual factors such as stereotype threat (Carr & Steele, 2010); Chinese are considered more risk seeking than Americans (Hsee & Weber, 1999); the nucleus accumbens is seen as influencing risk-seeking choices, whereas the anterior insula is seen as influencing riskless choices (Kuhnen & Knutson, 2005); high incentives are associated with more risk aversion than low incentives (Holt & Laury, 2005); and decision makers under high time pressure are seen as being more risk averse than decision makers under low time pressure (Zur & Breznitz, 1981). Likewise, stress is seen as affecting the amount of probability weighting in gains and losses (Porcelli & Delgado, 2009); the degree of striatal activity is assumed to influence the overweighting of small probabilities (Hsu, Krajbich, Zhao, & Camerer, 2009); framing the decision as involving precaution is assumed to lead to the overweighting of small and medium-sized probabilities (Kusev, van Schaik, Ayton, Dent, & Chater, 2009); age has been argued to generate more optimistic decision weights in gains (Pachur, Mata, & Hertwig, 2017); and decision feedback is considered to lead to linear probability weighting (Jessup, Bishara, & Busemeyer, 2008). Finally, it is often assumed that decision makers tend to weigh probabilities differently when gamble payoffs and probabilities are described compared with when these payoffs and probabilities are experienced (Hertwig, Barron, Weber, & Erev, 2004).

However, as we have shown, differences in modal choice proportions may be due to differences in the amount of variability in underlying parameters rather than to differences in central tendency parameter values. To illustrate, let us return to Figure 1. The horizontal axis shows a range of α*, and the vertical axis shows the choice probability for the risky gamble *X* corresponding with those different values of α*. The two lines reflect different levels of preference noise.

Now suppose we observe a male decision maker choosing *X* with frequency 0.53, whereas a female decision maker chooses *X* with frequency 0.47. If we were considering only the raw choice data, we might conclude that the male is somewhat risk seeking and the female is somewhat risk averse. If we allow for preference noise but suppose that the degree of such noise is the same for both individuals, we could still attribute the gap to different values of α*, with the male identified as being less risk averse. But if the male’s underlying preferences involve more preference noise (the dotted line) than the female’s (the solid line), we cannot draw that conclusion. Mapping from Pr[*X* chosen] = 0.53 via the dotted line gives the male’s α* as about 0.9, whereas mapping from Pr[*X* chosen] = 0.47 via the solid line gives the female’s α* as approximately 0.95, meaning that the male is, in terms of underlying preferences, actually more risk averse than the female.

The same point may hold for differences in best-fitting parameters across demographic groups or experimental conditions. If decision makers display both preference and response noise, but if the fitted model allows for only response noise, differences in the degree of preference noise across groups could be incorrectly interpreted as differences in underlying parameters. It is hard to say, in general, just how substantial any such effect might be: one could imagine it being stronger in some instances and weaker or insignificant in others. Our point is not to reject out of hand all of the differences reported in the studies cited earlier but rather to alert researchers to the possibility that the combination of parameter and response noise may in some cases lead to misestimates of the degree (and occasionally even the direction) of such differences.

## Nonlinearity in Parameters

In many ways, the above sections serve as an existence proof, showing how a combination of some types of preference and response noise can systematically distort choice probabilities. However, such effects are not limited to the particular functional forms of EUT and CPT or to the specifications of response and preference noise used in this paper. They are liable to apply whenever there is a nonlinear relationship between the parameters that describe preference and the utilities used to determine choice. In these circumstances, the means of the utility differences between options are liable to diverge from the utility differences generated by central tendency parameter values.

The supplemental materials examine various settings in considerable detail. However, to illustrate, we consider a different domain: intertemporal choice. Here the exponential discounting model (Frederick, Loewenstein, & O’Donoghue, 2002; Samuelson, 1937) is commonly used to model choices between rewards occurring at differing periods of time. One criticism of this model is that it cannot account for an increased preference for a proximate reward over a delayed reward because the lengths of the delay diminish by some common amount. For example, this model predicts that decision makers cannot prefer $10 in 3 months and 1 week to $5 in 3 months but also prefer $5 immediately to $10 1 week from now. Yet such present-biased choice patterns have often been reported, and they have been explained by alternative discount functions (see Frederick et al., 2002 for an overview). However, one could also explain such patterns using exponential discounting with preference noise. Indeed, if we use the exponential discounting model with a discount factor of δ = δ* + η_δ_, with δ* = 0.75, E[η_δ_] = 0 and η_δ_ uniform in [−0.25, 0.25] and if we assume (for simplicity) deterministic linear utility and a logistic response rule with θ = 1, we find that the probability of choosing $5 immediately over $10 in 1 week is 79% but that the probability of choosing $5 in 3 months over $10 in 3 months and 1 week is 48%. If we fail to allow for the role played by preference noise, this shift in modal choice may mislead us about the underlying time preferences of decision makers. Likewise, observed differences in intertemporal choice patterns that have been ascribed to differences in demographic, biological, neural, cognitive, emotion, social, and task-based factors may not exclusively reflect the impact of those factors on discount rates but might (to some extent, at least) reflect differences in the effects of noise.

## Appropriate Analysis

We have seen how the combination of preference and response noise can bias parameter estimates if model fits assume that underlying preferences are deterministic when in reality they are not.^11^ However, this does not mean that models such as EUT and CPT are unidentifiable in the statistical sense: parameters can be recovered accurately so long as the model is correctly specified.

Unlike current approaches to applying models like EUT and CPT (see, e.g., Broomell & Bhatia, 2014; Glöckner & Pachur, 2012; Harless & Camerer, 1994; Hey & Orme, 1994; Rieskamp, 2008; Stott, 2006), an appropriate analysis would assume a distribution over underlying parameter values. Such a hierarchical approach has been shown to be desirable for capturing group-level variability in the parameters of cognitive models of choice (Lee & Newell, 2011; Wetzels, Vandekerckhove, Tuerlinckx, & Wagenmakers, 2010; also see mixture logit models as in Train, 2009). Indeed, a recent study, using hierarchical Bayesian estimation (Nilsson, Rieskamp, & Wagenmakers, 2011; also Scheibehenne & Pachur, 2015), showed how this technique could be used to model prospect theory preferences without bias in settings with heterogeneous decision makers. Even though this work did not consider within-person preference noise, the statistical structure they proposed is essentially equivalent to the structure assumed in this paper, suggesting that hierarchical Bayesian estimation could be used to recover the parameters of CPT and EUT more accurately in the presence of preference noise at the individual level.

Of course, determining the correct model specification is not trivial. In this paper we have assumed a logistic model for response noise as well as an additive error model for preference noise. However, in reality this may not be the case. Indeed, other more sophisticated ways of modeling error have already been proposed (see Marley & Regenwetter, 2016 or Wilcox, 2008 for an overview), and many of these have desirable properties not possessed by the logit model. Currently we are unable to correctly diagnose the underlying noise specification based solely on data, and much more work needs to be done to identify appropriate error theories for modeling response and preference noise.

Another way to avoid the choice biases documented in this paper might involve the QTest method, which shows how choice proportions could be used to infer the underlying preferences of decision makers (Regenwetter et al., 2014; see also Regenwetter & Davis-Stober, 2012). This method attempts to characterize deterministic models with various stochastic assumptions in terms of the points on a multiple dimensional choice space that their choice predictions occupy. Currently this work appears to be applied either to deterministic models under the influence of response noise or else to random parameter specifications without response noise. However, we understand that the proponents of the QTest method are currently investigating the feasibility of combining these two sources of noise: if this is computationally tractable, it might provide a useful way to conceptualize the issues discussed in this paper.

A somewhat different line of development might build upon the true and error model (Birnbaum, 2013; Birnbaum & Bahara, 2012; Birnbaum & Diecidue, 2015). This approach assumes that decision makers have true preference orderings that are perturbed by errors but does not impose the kinds of functional restrictions assumed in our paper. Particularly, when presented with a number of choices within some block of questions in an experiment, an individual’s preferences are assumed to be consistent with some core (deterministic) theory throughout that block. However, the observed choices within that block may depart from that theory because of response error. In variants of this approach, it is also allowed that if the same choices are repeated in different blocks, the individual’s true preference might change from one block to another.

It may be possible to control for some of the effects outlined in this paper using the true and error framework and additionally use insights from this framework to identify necessary extensions and modifications of our own approach. For example, the experiments in Birnbaum (2013) suggest that preference noise is not independent and identically distributed, as assumed in this paper. Instead, there are cross-temporal correlations inherent in preferences. Indeed, one approach to modeling these correlations within a functional parametric structure has already been proposed by Birnbaum (2013, Appendix B), who suggests that parameters in utility functions can be seen to evolve according to a random walk process.

## Beyond Deterministic Models

We have used EUT and CPT to motivate and illustrate our arguments, but the issues we have discussed apply much more broadly. Although it has been known for more than half a century that human decision making is probabilistic (Mosteller & Nogee, 1951; Luce & Suppes, 1965), the evolution of modern decision theory has primarily involved the production of scores of deterministic models, whose developers have left the question of stochasticity in abeyance. When experimenters have tried to test these models, the most common strategy has been to add on some analytically convenient—but often rather arbitrary—error specification. However, it has become clear that the relationship between these deterministic models and their stochastic implementations is such that it is possible to drastically change inferences made using these models by altering assumptions regarding the nature of the variability in the data.

This problem is endemic to deterministic models of choice and cannot be fully remedied by the application of more rigorous methodological tools, even though the more recent techniques discussed in the previous section may represent improvements on simple logit specifications. We would argue that theoretical research on decision making should attempt to incorporate variability as part of the fabric of models rather than as ad hoc ways of giving a deterministic model a probabilistic appearance. There have already been a number of advances in modeling the cognitive basis of the stochastic choice process (Bhatia, 2013, 2014; Bogacz, Usher, Zhang, & McClelland, 2007; Busemeyer & Townsend, 1993; Diederich, 1997; Krajbich, Armel, & Rangel, 2010; Rangel & Hare, 2010; Roe, Busemeyer, & Townsend, 2001; Trueblood, Brown, & Heathcote, 2014; Tsetsos, Chater, & Usher, 2012; Usher & McClelland, 2004; see also Rieskamp et al., 2006 and Oppenheimer & Kelso, 2015 for useful discussions). Cognitive models of stochastic choice make explicit assumptions about how noise enters into deliberation and how it interacts with preference, choice, decision time, and confidence. In allowing stochasticity to play a central role in choice, these models are naturally able to capture a large range of behavioral effects that currently lie outside the descriptive scope of deterministic models. Indeed, some of these models even try to explain key decision-making anomalies using only unsystematic noise rather than specific restrictions on value functions or probability weighting (Bhatia, 2014; Navarro-Martinez, Loomes, Isoni, & Butler, 2014; also see, e.g., Ratcliff & Rouder, 1998). Moreover, experimental work has shown that these types of models outperform many of the deterministic utility models in terms of quantitative fit (Rieskamp, 2008). Future research should consider using these types of psychologically grounded choice models to understand the behavior of decision makers.

## Conclusion

We have shown that the coexistence of both preference noise and response noise—in each case modeled as zero-mean, symmetric, and independent—can systematically distort choice patterns. Thus, decision makers whose preferences are, on average, risk neutral, can display modal choice patterns that might be mistaken as evidence of risk aversion or risk seeking. Likewise, underlying preferences may be linear in probabilities but choice patterns may appear supportive of nonlinear probability transformations. In fact, a number of common and seemingly systematic decision anomalies can be generated by expected value maximizers with some degree of response and preference noise.

Our analysis suggests the need for care when trying to elicit the underlying preferences of decision makers. The presence of both preference and response noise can bias quantitative model fits if these fits do not make appropriate allowance for both sources of noise. Likewise, differences in choice proportions between various categories of decision makers may be due, at least to some extent, to different degrees of noise rather than being entirely attributable to intrinsic differences in preferences. In short, caution is needed when trying to infer the preferences of decision makers or when trying to identify the effects of psychological, biological, economic, and demographic variables on those preferences.

## Supplementary Material

10.1037/rev0000073.supp

## Footnotes

1 Numerous other functional forms are possible. Our conclusions do not depend on the particular choices of functional form, as we illustrate in the supplemental materials. Also note that evidence suggests that both EUT and CPT have some descriptive limitations (Birnbaum, 2008; Starmer, 2000). However, our primary purpose is not to defend or advocate either theory; we simply take them as two examples of risky utility maximization models and explore the difficulties of inferring their parameters and distinguishing their predictions in the presence of both preference and response noise.

2 Although we present Luce’s and Thusrtone’s approach as being closely related, they do have diverging interpretations. Choice in Thurstone’s theory is often seen as involving the comparison of random variables or discriminable processes. In case 5 of this theory, these random variables involve scale values (such as *U*(*X*|α,γ) and *U*(*Y*|α, γ)) combined with additive errors (such as ε). In contrast, Luce derives his theory from axiomatic restrictions on choice probabilities (see Marley & Regenwetter, 2016, or Wilcox, 2008, for an overview). There are also other related approaches that depart from the assumption that response noise is a function of only the utility differences (Carroll, 1980; Restle, 1961; Wilcox, 2011). For simplicity, we will not be explicitly examining the predictions of these approaches in this paper, although we do discuss them briefly in the supplemental materials.

3 This approach is closely related to models of stochastic ordering (Block & Marschak, 1960; Regenwetter, Dana, & Davis-Stober, 2011; Regenwetter & Marley, 2001). These models involve relational preferences, which are drawn at random, so that a decision maker chooses *X* or *Y* based on whether *X* ≻ *Y* or *Y* ≻ *X* is sampled in any given trial (typically without any additional response noise or error). If variability in parameters were the only source of noise in the decision, then our above specification could be represented in terms of a random ordering.

4 In the supplemental materials, we provide additional analysis demonstrating corresponding distortions for a variety of different gambles.

5 The other two aspects of the 4-fold pattern pertain to the loss domain, in which the mirror-image pattern is generated by the interaction between the probability weighting function and a loss function, which exhibits diminishing sensitivity.

6 The supplemental materials examine the robustness of this result, including the implications for Tversky and Kahneman’s (1992) 4-fold pattern gambles.

7 In the supplemental materials, we show that this effect also emerges for Kahneman and Tversky’s (1979) classic common ratio gambles.

8 Specifically, we take α* values in the set (0.5, 0.6, 0.7, 0.8, 0.9, 1.0, 1.1, 1.2, 1.3, 1.4, 1.5), which implies that α varies between 0 and 2 in the simulations. Response noise in utilities is generated by a logit choice function with θ = 1. For each value of α*, the corresponding EUT model is simulated 1,000 times on Stott’s 90 gamble pairs. For each gamble in each simulation, the model’s choice is sampled according to the choice probability in Equation 2. Again note that the same sampled value of α is used for both options within a choice, but α varies across choices.

9 In this recovery, we approximate the continuous uniform distribution over parameter values present in data-generating model (i.e., η uniform in [−0.5, 0.5]) with a discrete uniform distribution (η uniform over the set [0.5, 0.6, 0.7, 0.8, 0.9, 1.0, 1.1, 1.2, 1.3, 1.4, 1.5]). This is done for computational tractability. However, it does mean that our data-generating model is not perfectly identical to the fitted model. Ultimately, the mean squared error for the fits obtained as part of this recovery is an upper bound for the *MSE* that could be expected if the fitted model was perfectly specified. Also note that this recovery assumes that the variance in parameters (i.e., η_α_ distributed uniformly in the interval [−0.5, 0.5]) is known. A more complex recovery that also estimates parameter variance should also be able to recover true parameters as already shown by Nilsson et al. (2011) and others.

10 Model fits are performed by maximizing log likelihood and are implemented in MATLAB using the simplex routine, with starting points for each of the fits set at α_*start*_ = α* and θ_*start*_ = 1. The use of these starting points ensures that any divergence between the true underlying parameters and the recovered parameters is not due to our fits converging on suboptimal local maxima.

11 Note that the alternative type of misspecification also leads to incorrect inferences. If decision makers have both preference and response noise but it is (incorrectly) assumed that they have only preference noise, inferences made using either modal choices or model fits can be biased.
