# Supplementary material for: Noisy Preferences in Risky Choice: A Cautionary Note
Source: Psychol Rev. 2017 Jun 1;124(5):678–87. doi: 10.1037/rev0000073 (PMC5619393; doi:10.1037/rev0000073)
Supplement: Supplementary file 1 [file z2q999172501so1.docx]

**Supplemental Materials**

**Noisy Preferences in Risky Choice: A Cautionary Note**

**by S. Bhatia & G. Loomes, 2017, *Psychological Review***

**http://dx.doi.org/10.1037/rev0000073**

**Qualitative Inferences**

In this document we present further analysis demonstrating the effect of preference noise and response noise on choice probabilities and the inferences made from choice data. In this section, we examine qualitative inferences made about risk preferences from modal choice probabilities. In the main text of this paper we find that decision makers displaying risk averse underlying preferences (*α** < 1 with *γ* fixed at *γ* = 1) can generate higher choice probability for simple risky gambles compared to their expected values, a behavior typically seen to be indicative of risk seeking. To examine the robustness of this effect we randomly generated *N* = 1,000 risky gambles *X* = (*x_1_*, *p*_1_; *x_2_*, 1 - *p*_1_) with *x_1_* and *x_2_* drawn from a uniform distribution with range [0, 25], and *p*_1_ drawn from a uniform distribution with range [0, 1]. For these 1,000 gambles we also generated their expected value equivalents *Y* = (*p*_1_∙ *x_1_* + (1-*p_1_*) ∙*x_2_*, 1), and examined the proportion of times a decision maker with risk averse EUT preferences would choose the risky option *X* over its expected value *Y*. As above we modelled response noise with a logit function with *θ* = 1, and preference noise with *α** = 0.9 and *η_α_* distributed uniformly in the interval [-0.5, 0.5], and found that the model with both types of noise chose the risky option with an average probability of 0.52 (median = 0.51, *SD* = 0.02). Again, this is despite the fact that the central tendency of the decision maker’s underlying preferences correspond to risk aversion. By contrast, for an identical model without preference noise, the probability of choosing the risky gamble would always lie below 0.5 regardless of the specific randomly generated choice problem it is applied to.

The main text of the paper also found a related result for the fourfold pattern of choice. Particularly, it showed that a decision maker with risk neutral linear probability weighting could more frequently choose low-probability risky gambles and more frequently choose high-probability risky gambles (relative to their respective expected values). Again we examine the robustness of this type of distortion. For this purpose we considered the set of seven two-outcome gambles^[[1]](#footnote-1)^ used by Tversky and Kahneman (1992) to illustrate the fourfold pattern for gains. In their experiment, Tversky and Kahneman varied the probability of the larger outcome in the gamble between 0.01 and 0.99 and tested the effect of changing this probability on the certainty equivalents for the gambles provided by the participants. Consistent with the hypothesised fourfold pattern, they found that their participants were predominantly risk averse (certainty equivalent lower than the expected value of the gamble) when the probabilities were high, but risk seeking (certainty equivalent higher than the expected value) when the probabilities were low.

We tested the effect of both preference and response noise on the choices between these gambles and their certainty equivalents. We set *α* = 1 and *γ*^*^ = 1, and allowed *η_γ_* to vary uniformly in the interval [-0.5, 0.5]. For response noise, we used the logit function with *θ* = 1. As in Tversky and Kahneman’s experiment we found that our model was more likely to select the gambles over their expected value when the probabilities of the larger outcomes were low (indicating risk seeking for small probabilities), but less likely to do so when these probabilities were high (indicating risk aversion for large probabilities). This is despite the fact that *γ*^*^ = 1, meaning that the central tendency is for linear probability weighting (in addition to the risk neutrality entailed by *α* = 1). If the model’s parameters were not variable, it would always give a 50% chance of choosing the gamble over its expected value. Figure S1 displays the choice probabilities generated by the above model for the seven different gambles and nine outcome probabilities used by Tversky and Kahneman.^[[2]](#footnote-2)^

Finally, we examine the common ratio effect. Again, in the main text we showed how this effect could be generated by risk neutral decision makers with linear utility. In our demonstration we used artificial gambles *X^III^*, *Y^III^*, *X^IV^*, and *Y^IV^* to ensure that the expected values of the gambles in any given choice problem are identical. This makes it easier to tease out the effects of preference noise, as without this noise the choice probabilities should always be 50% (as *α* = 1 and *γ** = 1). However, it would also be useful to replicate the above analysis with Kahneman and Tversky’s (1979) classic common ratio gambles. These gambles^[[3]](#footnote-3)^ are *X_KT_^III^* = (4, 0.8; 0, 0.2), *Y_KT_^III^* = (3, 1), *X_KT_^IV^* = (4, 0.2; 0, 0.8), and *Y_KT_^IV^* = (3, 0.25; 0, 0.75). For each pair of these gambles we compare the choice probability of the risky option over the safe option generated by a model with *α* = 1, *γ*^*^ = 1 and no preference noise to the analogous choice probability generated by a model with *α* = 1, *γ*^*^ = 1 and *η_γ_* uniform in [-0.5, 0.5]. As expected we find that permitting preference noise decreases the choice probability of *X_KT_^III^* (from 0.55 with preference noise to 0.54 without preference noise) but increases the choice probability of *X_KT_^IV^* (from 0.512 to 0.514), indicating that the presence of preference noise shifts choice patterns in a manner that corresponds to observed shifts in choice behavior. A similar test with Kahneman and Tversky’s (1979) common consequence gambles *X_KT_^III^* = (2.5, 0.33; 2.4, 0.66, 0, 0.01), *Y_KT_^III^* = (2.4, 1), *X_KT_^IV^* = (2.5, 0.33; 0, 0.67) and *Y_KT_^IV^* = (2.4, 0.34; 0, 0.66) also gives us this result. Preference noise decreases the choice probability of *X_KT_^III^* (from 0.502 to 0.499) but increases the choice probability of *X_KT_^IV^* (from 0.502 to 0.503), once again showing that the presence of preference noise shifts choice patterns in a manner that corresponds to observed shifts in choice behavior.

**Robustness**

**Functional forms.** To what extent are the above results dependent on our choice of a power value function and the Prelec (1998) probability weighting function? Not greatly. The primary cause of the effects documented above is the non-linear manner in which response noise enters into the utility function. As long as the value and probability weighting functions under consideration are non-linear in their parameters (and they almost always are), we would expect variability in these parameters to interact with response noise and distort choice probabilities. Additionally, as long as the influence of these parameters on the general shapes of these functions is similar to the influence of *α* and *γ* on the power value function and Prelec probability weighting function, the direction and magnitude of the effect of variability in these parameters should approximate the results documented above.

Consider, for example, replacing the Prelec (1998) probability weighting function with single parameter versions of the probability weighting function used by Tversky & Kahneman (1992) (Equation S1) or the probability weighting function used by Gonzalez & Wu (1999) (Equation S2):

$w\left( p_{1} \right)= \frac{{p_{1}}^{\gamma}}{{({p_{1}}^{\gamma}+\left( 1-p_{1} \right)^{\gamma})}^{1/\gamma}}$ (S1)

$w\left( p_{1} \right)= \frac{{p_{1}}^{\gamma}}{{p_{1}}^{\gamma}+\left( 1-p_{1} \right)^{\gamma}}$ (S2)

These two functions are similar to Prelec’s (1998) function (presented in Equation 1 of the main text): Both display an overweighting of small probabilities and underweighting of large probabilities for *γ* < 1, and an overweighting of large probabilities and underweighting of small probabilities for *γ* > 1. At *γ* = 1 they are linear. Due to these similarities we would expect the results documented above, for varying *γ*, to persist if Prelec’s function is replaced by Tversky and Kahneman’s or Gonzalez and Wu’s function.

We consider the fourfold pattern of choice, with gambles *X^I^* = (10, 0.01; 0, 0.99), *Y^I^* = (0.10, 1), *X^II^* = (10, 0.99; 0, 0.01), and *Y^II^* = (9.90, 1), to test this intuition. Again we fix *θ* = 1, *α* = 1, and *γ^*^* = 1 and allow *η_γ_* to be uniform in [-0.5, 0.5]. With this specification we obtain Pr[*X^I^* chosen] = 0.52 and Pr[*X^II^* chosen] = 0.45 for Tversky and Kahneman’s function, and Pr[*X^I^* chosen] = 0.53 and Pr[*X^II^* chosen] = 0.47 for Tversky and Kahneman’s function. This is very similar to Pr[*X^I^* chosen] = 0.54 and Pr[*X^II^* chosen] = 0.47, which is what we obtained using Prelec’s function. Thus preference noise can generate risk seeking for small probabilities and risk aversion for large probabilities (in gains), despite the central tendency of the decision maker’s preferences corresponding to linear probability weighting and linear value, even if we replace our initial probability weighting function with other well-known weighting functions. Similar results hold for the other fourfold pattern gambles and the common ratio gambles. Additionally, we would expect the findings relating to risk aversion and risk seeking to persist if the power value function is replaced by a different value function with similar parametric properties.^[[4]](#footnote-4)^

**Amount of preference noise.** Another determinant of the strength of the above results is the extent of the variability of the parameters. In the main text we considered examples where *α* or *γ* involved *η_α_* or *η_γ_* uniform in [-0.5, 0.5]. However, the error terms *η_α_* and *η_γ_* could be drawn from many other distributions. In general, more variance in *η_α_* and *η_γ_* leads to a larger effect of preference noise, and thus increased impacts upon choice probability. This happens because, keeping the central tendency constant, greater variance typically increases the distance between the average utility difference and the utility difference when the parameter takes its mean value.^[[5]](#footnote-5)^

As an example, consider again the fourfold pattern, with a fixed *θ* = 1, *α* = 1, and *γ^*^* = 1 (applying to Prelec’s probability weighting function). Additionally, let *η_γ_* be uniform in [-*k*, *k*], with *k* varying in the interval (0,1). A larger value of *k* indicates increased noise in *γ*, and the above reasoning would suggest that larger values of *k* would lead to higher Pr[*X^I^* chosen] and lower Pr[*X^II^* chosen] (corresponding to stronger distortions in choice probability). Figure S2 supports this prediction. Small values of *k*, corresponding to reduced preference noise, generate Pr[*X^I^* chosen] ≈ Pr[*X^II^* chosen] ≈ 0.5, whereas large value of *k*, corresponding to increased preference noise, lead to Pr[*X^I^* chosen] >> 0.5 >> Pr[*X^II^* chosen]. Indeed, for *k* = 0.99, we find that Pr[*X^I^* chosen] = 0.61 and Pr[*X^II^* chosen] = 0. 39, which is notably larger than Pr[*X^I^* chosen] = 0.54 and Pr[*X^II^* chosen] = 0.47 obtained in our earlier analysis (for which *k* = 0.5). Similar results hold for the other fourfold pattern gambles, common ratio gambles, and risk preference gambles, and other symmetric distribution functions for the noise terms. In fact, we would expect this effect to persist in nearly all settings in which preference noise plays a role.

**Utility differences.** Two other factors are the magnitude of the outcomes offered in the gambles and the degree of response noise at play in the decision. These two factors are closely related, as they both influence the way utility differences map onto choice. In fact, both of these variables influence choice probabilities even in the absence of response noise. With preference noise, however, they also influence the strength of the observed choice probability distortions.

First, consider the role of gamble outcomes. If the magnitude of these outcomes is increased uniformly across the gambles (by, for example, multiplying all relevant outcomes by a large positive constant), then the differences between gamble utilities will also tend to grow larger, which in turn is liable to widen the gap between the average utility difference and the utility difference when the parameters are set at their mean values. If all else is held constant, this can exacerbate the distortion generated by preference noise. Consider again our fourfold pattern example. As with the above analysis, we fix *θ* = 1, *α* = 1, and *γ^*^* = 1, allow *η_γ_* to be uniform in [-0.5, 0.5], and apply our model with Prelec’s probability weighting function. Additionally, we use gambles *X^I^* = (*k∙*10, 0.01; *k∙*0, 0.99), *Y^I^* = (*k∙*0.10, 1), *X^II^* = (*k∙*10, 0.99; *k∙*0, 0.01), and *Y^II^* = (*k∙*9.90, 1), which are identical to the gambles used in the above analysis, but with a multiplier, *k*, on the gamble outcomes. As discussed above, we obtain Pr[*X^I^* chosen] = 0.54 and Pr[*X^II^* chosen] = 0.47 when *k* = 1. This changes to Pr[*X^I^* chosen] = 0.60 and Pr[*X^II^* chosen] = 0.41 when *k* = 10, and to Pr[*X^I^* chosen] = 0.504 and Pr[*X^II^* chosen] = 0.497 when *k* = 0.1, indicating that larger gamble outcomes lead to increased changes in choice probability (relative to the setting without preference noise in which Pr[*X^I^* chosen] = Pr[*X^II^* chosen] = 0.5).

It is important to note, however, that this effect is not monotonic, so that very large gamble outcomes can reduce the observed distortions. For example, if we consider *k* = 100, then with the above examples we obtain Pr[*X^I^* chosen] = 0.502 and Pr[*X^II^* chosen] = 0.496, which is a much weaker distortion compared to *k* = 10 and *k* = 1. This happens due to the magnitude of the response noise at play in the decision. For moderate values of *θ* (such as *θ =* 1), utility differences for small and moderately sized gamble outcomes (such as *k* = 1 or *k* = 10), lie in the roughly linear region of the logistic response function. Here the divergence between the expected utility difference given the parameters and the utility difference for the mean parameters has the strongest impact, and choice probabilities display the greatest distortion. For this reason, small increases to the magnitude of these outcomes, and correspondingly, small increases in the utility differences, typically increase the distortions in the final choice probabilities. In contrast, for very large gamble outcomes, such as with *k* = 100, utility differences are also large, and lie at the non-linear edges of the logistic response function. In these regions, choice probability is largely flat for both highly positive utility differences and for highly negative utility differences, indicating that increasing utility differences does not substantially increase probability distortions, and can in fact reduce probability distortions. This is why the effect of *k* on the above distortions is non-monotonic.

The degree of response noise at play in the decision has a similar effect on the magnitude of observed probability distortions. While increasing gamble outcomes can increase utility differences, decreasing the amount of response noise can make given utility differences more impactful. For moderate amounts of response noise, combined with moderate utility differences, decreasing the amount of response noise typically increases the distortion in observed choice probabilities. However, when there is little response noise (or when utility differences are very large) further reductions to the amount of response noise can reduce or even eradicate observed probability distortions.

Again consider the gambles *X^I^* = (10, 0.01; 0, 0.99), *Y^I^* = (0.10, 1), *X^II^* = (10, 0.99; 0, 0.01), and *Y^II^* = (9.90, 1), setting *α* = 1, *γ^*^* = 1, and *η_γ_* uniform in [-0.5, 0.5]. As outlined above, we obtain Pr[*X^I^* chosen] = 0.54 and Pr[*X^II^* chosen] = 0.47 when *θ* = 1. This changes to Pr[*X^I^* chosen] = 0.60 and Pr[*X^II^* chosen] = 0.41 when *θ* = 10, to Pr[*X^I^* chosen] = 0.504 and Pr[*X^II^* chosen] = 0.497 when *θ* = 0.1, and to Pr[*X^I^* chosen] = 0.502 and Pr[*X^II^* chosen] = 0.496 when *θ* = 100. Clearly changing the magnitude of *θ* has an identical effect as changing the magnitude of *k*. This is to be expected, as with *α* = 1 and a logit choice rule, *k* and *θ* have identical linear interactions with the size of the utility difference.

The above results have been demonstrated with a logit response rule related to Luce’s (1959) model. However, the underlying insights also hold for other strong utility models, such as the probit choice rule, which relies on normally distributed response noise (Thurstone, 1927). As with the logit rule, choice probability is increasing in the magnitude of utility difference. This relationship is roughly linear for small utility differences and for large amounts of response noise but nonlinear for larger utility differences and intermediate amounts of response noise. Thus, as above, we would expect preference variability-based choice probability distortions to display a non-monotonic relationship with utility difference and response noise when response noise is formalized using the probit model.

These results can be expected to hold for a number of moderate utility models of response noise. Such models typically amplify or diminish utility differences based on features of the choice context, such as the similarity between the gambles or the range of utilities and outcomes at play in the decision (Carroll, 1980; Wilcox, 2011). If these features are held constant, many of these models can be described using logit or probit-style response rules (with appropriate values of parameters such as *θ*). If, in contrast, these feature vary from one set of gambles to another, then the effect of changing the gamble context can be understood in terms of its effect on the change in the impact of relative utility differences.

Consider, for example, the contextual utility model (Wilcox, 2011), which divides utility differences of a pair of gambles by the difference between the maximum utility and minimum utility obtainable in a given choice context (such as in an experimental session). In a setting where gamble outcomes range between $0 and $10, utility differences would be normalized by the difference between the utility of $10 and the utility of $0. In contrast, in a setting where gamble outcomes range between $0 and $100, utility differences would be normalized by the difference between the utility of $100 and the utility of $0. In both settings, the maximum utility for a gamble would be assigned a value of 1 and thus the maximum utility difference (in the gain domain) would be scaled to 1. When embedded in a logit response rule, preference-noise-based choice distortions in such a model would depend largely on the chosen value of *θ*, as the contextual normalization term would attenuate the effect of changes in outcome magnitudes. Thus effects generated by preference noise would become more robust, and would emerge regardless of the magnitude of rewards at play in the choice task.

**Three-Option Choice.** Our results extend beyond binary choice to settings with three or more gambles. Here, response noise is often modelled using the multinomial logit and probit models (e.g. McFadden, 1973). The former is a straightforward extension of the logit rule outlined in the main text: it merely replaces the denominator in Equation 2 with the sum of the exponentials of the utilities of all the gambles under consideration.^[[6]](#footnote-6)^ Likewise, the latter permits all the gambles to have normally distributed error terms, and defines the choice probability of any particular gamble in terms of the probability that its utility, combined with its add-on error, exceeds the utility plus error realizations for all other gambles. In both settings the choice probability of a given gamble is increasing in its utility difference relative to the other gambles. For this reason, as in the examples discussed in this paper, the interactive effect of preference and response noise can be understood in terms of the deviation between the mean of the differences between utilities and the utility differences derived from the mean values of the parameters. Thus in multiple choice settings as in binary choice settings we can expect effects that limit our ability to use modal choices to infer underlying preferences.

**Quantitative Inferences**

In the main text of the paper we examined the effects of preference noise on quantitative inferences about *α* and *γ* separately. Thus in the first parameter recovery study we allowed for variability in *α* while keeping *γ* = 1 in both our fits and our recovery (and vice versa in our second parameter recovery study). However, most CPT model fitting studies attempt to recover both parameters simultaneously. So here we simulate such a parameter recovery study. Our methods are identical to those used in the main text, except that both *α** and *γ** are allowed to independently take values in the set {0.5, 0.6, 0.7, 0.8, 0.9, 1.0, 1.1, 1.2, 1.3, 1.4, 1.5}, leading to a total of 11x11 = 121 different combinations. We run two sets of simulations: one in which *α* and *γ* are both deterministic and there is only response noise; and one in which we also allow for noisy *α* and *γ*, with *η_α_* and *η_γ_* distributed uniformly in the interval [-0.5, 0.5]. We recover best-fit values of *α* and *γ* (as well as *θ*) using the same three recovery methods as before and compare these with the values of *α** and *γ** that generated the data.

Figures S2a and S2b display the median recovered *α_fit_* and *γ_fit_* for each value of *α** and *γ** for the three sets of recoveries. In obtaining *α_fit_* for each *α**, we pool recovered values of *α* obtained over all of the varying values of *γ**. Likewise, in obtaining *γ_fit_* for each *γ**, we pool recovered values of *γ* obtained over all of the varying values of *α**. These figures display the same patterns as the figures in the main text. Particularly *α** and *γ** are recovered successfully when the fitted model is correctly specified (the second and third recovery) but are systematically distorted when model being fit is misspecified (first recovery). Overall, the mean-squared errors for *α­_fit_* and *γ_fit_* are 131.03 x 10^-4^ and 34.69 x 10^-4^ for the first recovery, 34.51 x 10^-4^ and 16.04 x 10^-4^ for the second recovery, and 8.75 x 10^-4^ and 5.41 x 10^-4^ for the third recovery. Additionally, we find that *α­_fit_* and *γ_fit_* both deviate from *α** and *γ** in the same ways as we saw earlier. Thus decision makers for whom 1 ≤ *α** ≤ 1.2 may appear to be risk averse (*α_fit_* < 1); and individuals for whom *γ** = 1 appear as if they overweight small probabilities (*γ_fit_ <* 1).


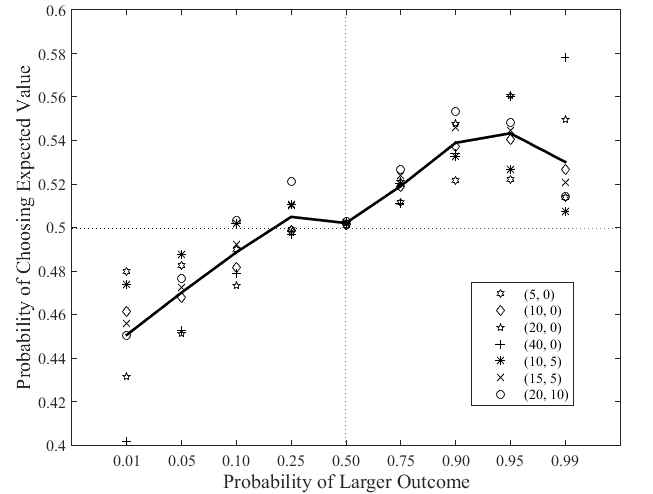


*Figure S1*. The fourfold pattern for Tversky and Kahneman’s (1992) gambles. Here we plot the probability of choosing the expected value of the gamble over the gamble itself as a function of the probability of the larger outcome of the gamble. The legend indicates the outcomes of the corresponding gambles and the solid line plots the average choice probability for the seven gambles for the probability value in consideration. Here we have *θ* = 1, *α* = 1, *γ^*^* = 1, and *η_γ_* uniform in [-0.5, 0.5].


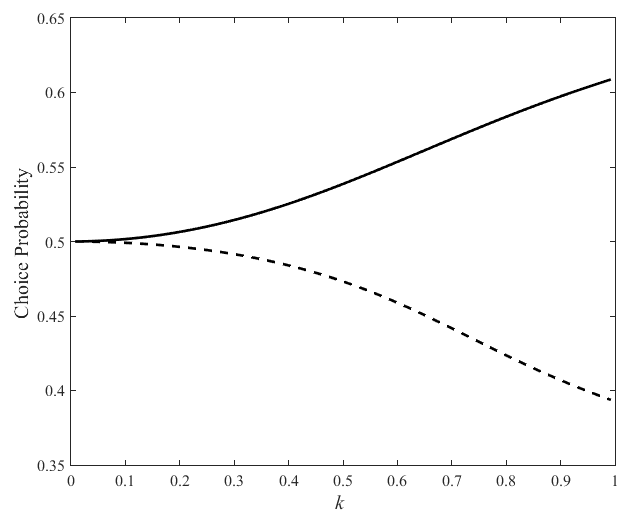


*Figure S2.* The probability of choosing a low-probability risky gamble *X^I^* over its expected value *Y^I^* (solid line) and the probability of choosing a high-probability risky gamble *X^II^* over its expected value *Y^II^* (dashed line) as a function of *k*. Here we have *θ* = 1, *α* = 1, *γ^*^* = 1, and *η_γ_* uniform in [-*k*, *k*].


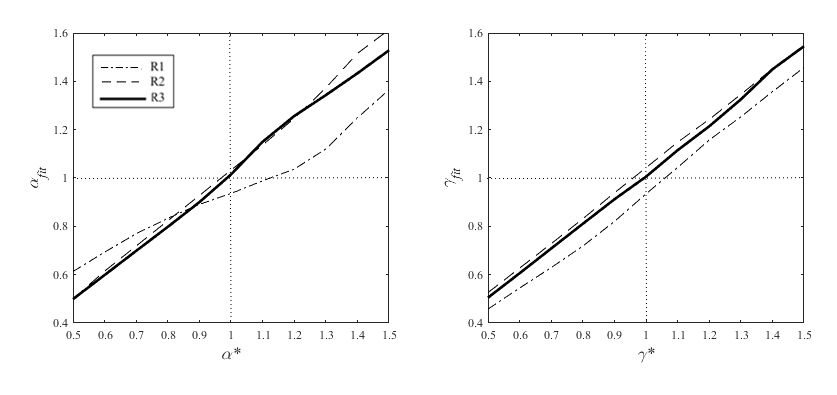


*Figures S3a and S3b.* Median recovered values of *α*, *α_fit_*, plotted against *α^*^* (left panel) and median recovered values of *γ*, *γ_fit_*, plotted against *γ^*^* (right panel). The first parameter recovery (R1) involves a data generating model with both response and preference noise and misspecified fitted model which assumes only response noise. The second parameter recovery (R2) involves a data generating model and correctly specified fitted model with both response and preference noise. The third parameter recovery (R3) involves a data generating model and correctly specified fitted model with only response noise. Unlike the main text this recovery estimates both parameters together.

1. Note that for current purposes we divide the outcomes of these gambles by ten so that the outcomes lie between 0 and 40 rather than 0 and 400 (similar in magnitude to the other gambles examined in this paper). [↑](#footnote-ref-1)
2. The results presented in this section also extend to the loss domain, where modal choices display the opposite pattern. This reversal is due to the assumption of diminishing sensitivity in losses, and this assumption can be combined with the type of preference noise studied in this section, to generate the choice patterns observed in decision makers. [↑](#footnote-ref-2)
3. Again we scaled down these gambles by a factor of 1000 so that their outcomes are similar in magnitude to the gambles examined in this paper [↑](#footnote-ref-3)
4. However, note that the power value function used in this paper is particularly flexible in being able to capture risk aversion, risk neutrality, and risk seeking, with different values of the same parameter (*α* > 1, *α* = 1, or *α* < 1). In contrast, logarithmic value and exponential value, two other popular functions, always generate either risk aversion or risk seeking. [↑](#footnote-ref-4)
5. All else equal, if the convexity or concavity in this relationship is non-decreasing, larger variability in *η_α_* and *η_γ_* would lead to a larger gap between the expected utility difference and the utility difference of the expected parameter, creating a stronger bias. [↑](#footnote-ref-5)
6. Though the relationship between the multinomial logit and multinomial probit is not so straightforward in non-binary settings (Yellott, 1977). [↑](#footnote-ref-6)
